# Visualization of Microcirculation Using GOKO Bscan-ZD in a Patient with Chronic Limb-Threatening Ischemia: A Case Report

**DOI:** 10.3400/avd.cr.26-00061

**Published:** 2026-08-28

**Authors:** Takafumi Fujita, Makoto Sugihara, Kaori Mine, Tetsuo Hirata, Yasunori Suematsu, Takashi Kuwano, Shin-ichiro Miura

**Affiliations:** Department of Cardiology, Fukuoka University School of Medicine, Fukuoka, Fukuoka, Japan

**Keywords:** microcirculation, chronic limb-threatening ischemia, LDL apheresis

## Abstract

A 50-year-old man with type 2 diabetes mellitus was admitted for chronic limb-threatening ischemia (CLTI). Ulcers involved several toes on both feet. Angiography of the left lower extremity demonstrated severe below-the-ankle arterial disease with markedly compromised pedal runoff. After unsuccessful endovascular therapy of the left lower extremity, low-density lipoprotein (LDL) apheresis was initiated for limited-option CLTI. An increase in microcirculatory flow in the left foot was observed after LDL apheresis, as assessed by angiography and GOKO Bscan-ZD. Complete wound healing was achieved after a transmetatarsal amputation. GOKO Bscan-ZD may represent a novel noninvasive method with which to evaluate microcirculation.

## Introduction

Low-density lipoprotein (LDL) apheresis therapy (Rheocarna; Kaneka, Osaka, Japan) is utilized in Japan as an adjuvant treatment for patients with chronic limb-threatening ischemia (CLTI) after revascularization, and may be covered by Japan’s National Health Insurance in such cases. LDL apheresis therapy removes LDL cholesterol and fibrinogen—factors that determine plasma viscosity—from the bloodstream.^1)^ Improved hemorheology and microcirculation are drivers for efficient wound healing. Soga et al.^2)^ reported that LDL apheresis therapy, described as a “viscosity intervention,” improved microcirculation. Furthermore, it has been reported that LDL apheresis improved the angiographic speed of arterial blood flow and allowed for the visualization of venous return.^2,3)^ These findings are indicative of a positive response to viscosity intervention in regard to wound healing.

As macrovascular patency does not always translate to adequate tissue perfusion, the evaluation of microcirculation is essential for predicting wound healing in patients with CLTI. Skin perfusion pressure (SPP) and transcutaneous oxygen tension are commonly used to assess tissue perfusion in patients with CLTI,^4)^ as the results of these examinations may be strong predictors of wound healing.^5)^ However, in clinical practice, SPP measurements are often incorrect owing to pain or involuntary patient movement.

GOKO’s Bscan-ZD capillaroscope (GOKO Imaging Devices, Kanagawa, Japan) may represent a novel method for evaluating microcirculation. Capillaroscopy is performed on a patient’s fingers, toes, and dorsalis pedis using an optical probe at high (×720) and low (×200) magnifications (**Supplementary Fig. 1**). This noninvasive test does not cause pain and can be performed anywhere, such as in outpatient clinics or catheterization laboratories, if the appropriate equipment is available. Capillaroscopy has previously been used to assess the efficacy of treatments in patients with Raynaud’s phenomenon and lymphedema.^6,7)^ We report herein a case in which microcirculatory changes were observed after LDL apheresis in a patient with CLTI, as visualized using a GOKO Bscan-ZD in conjunction with dedicated software (GOKO-VIP; GOKO Imaging Devices), which measured the flow velocity of the microcirculation.

## Case Report

**Fig. 1:**
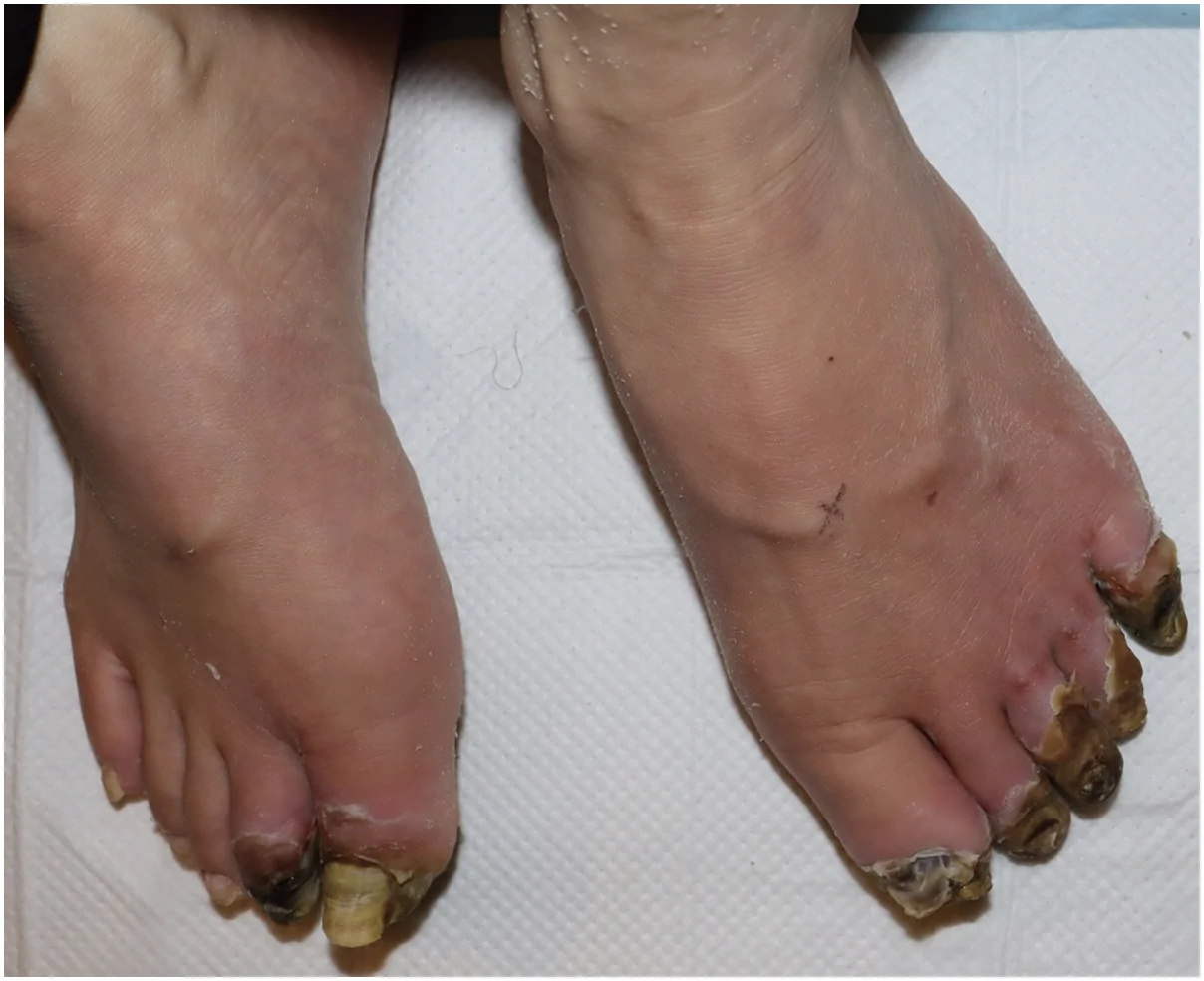
Ulcers of several toes.

**Fig. 2:**
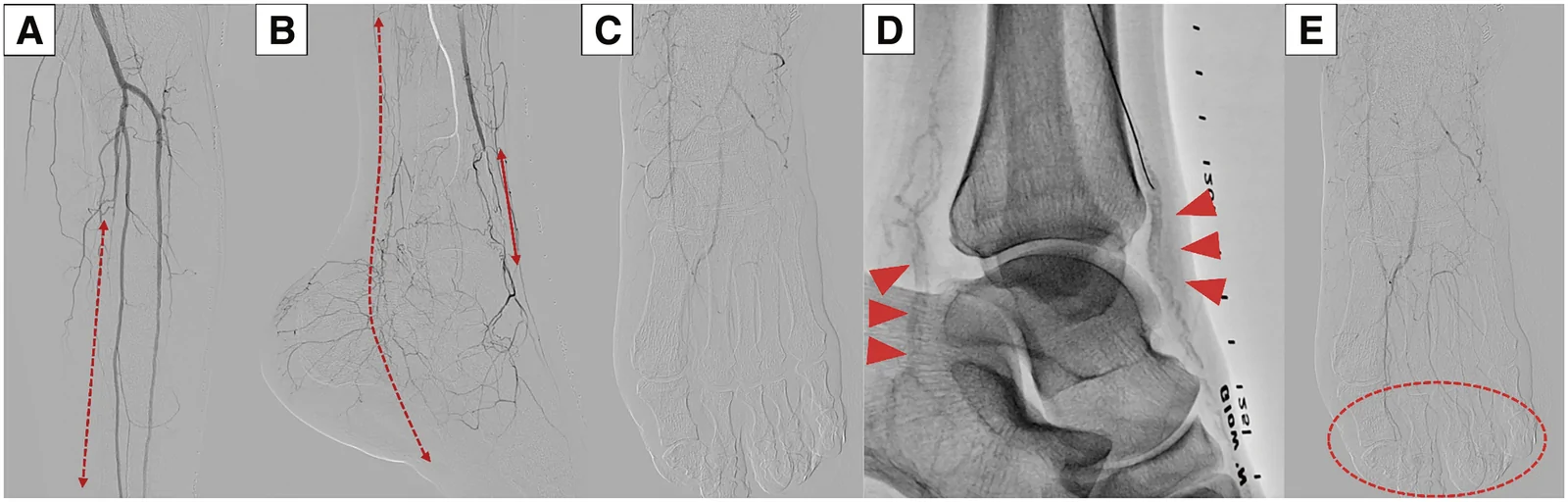
Baseline angiography. (**A**) Digital subtraction angiography showing occlusion of the left posterior tibial artery (dotted line). (**B**) Digital subtraction angiography showing occlusion from the left posterior tibial artery to the plantar artery (dotted line), indicative of occlusion of the left dorsal artery (solid line). (**C**) Initial angiography of the dorsalis pedis. (**D**) Medial calcifications in the below the ankle arteries (arrowheads). (**E**) Digital subtraction angiography showing the dorsal metatarsal arteries after LDL apheresis; the dotted circle indicates wound blush. LDL, low-density lipoprotein

A 50-year-old man with type 2 diabetes mellitus was admitted to our hospital for the treatment of CLTI. The ulcers were localized in several toes of both lower extremities (**Fig. 1**). The femoral and popliteal arteries were palpable; however, the dorsal and plantar arteries were not. He had no history of catheter-based arterial intervention before admission. Laboratory tests on admission revealed a C-reactive protein level of 0.35 mg/dL, a white blood cell count of 7300/μL, an eosinophil count of 0.8%, a serum creatinine level of 0.64 mg/dL (estimated glomerular filtration rate, 100.6 mL/min/1.73 m^2^), a serum albumin level of 3.6 g/dL, an LDL cholesterol level of 73 mg/dL, and a hemoglobin A1c level of 7.5%. The patient’s left ankle–brachial index was 1.12. The SPP measured at his left dorsalis pedis and plantar regions were 52 and 42 mmHg; however, the result was not accurately obtained because of pain. The Wound, Ischemia, foot Infection (WIfi) classification of the patient’s left lower limb was wound = 2, ischemia = 1, and foot infection = 0, corresponding to a clinical stage of 3. Contrast-enhanced computed tomography (CT) performed before admission showed no apparent shaggy aorta or severely atheromatous plaques in the abdominal aorta, and no significant lesions in the iliac segments (**Supplementary Video 1**). Based on these physical findings, we considered the possibility of a refractory ulcer due to lower limb ischemia. Initial angiography revealed no significant lesions in the femoropopliteal segments, although the posterior tibial artery was occluded from the proximal segment. (**Fig. 2A**). Additionally, the distal anterior tibial artery was occluded (**Fig. 2A** and **2B**), and the dorsalis pedis artery was visualized only via collateral circulation, indicating severely compromised pedal runoff (**Fig. 2B** and **2C**). A distal bypass was considered; however, adequate pedal target vessels could not be identified because of severely compromised pedal circulation and poor runoff. We attempted endovascular therapy (EVT) for the lesions in the below-the-knee (BK) arteries; however, the guidewire could not be advanced because of the severely calcified lesions (**Fig. 2D**). After multidisciplinary discussion within the foot care team, transmetatarsal amputation was considered the most appropriate treatment option for the left lower extremity. However, given the severely compromised BK arterial status, we decided to prioritize treatment of the left lower extremity first. Because EVT was technically unsuccessful, LDL apheresis was subsequently initiated to promote wound healing in the left lower extremity. In contrast, the right foot ulcers were managed conservatively during the present hospitalization, and treatment of the right lower extremity was deferred until a subsequent hospitalization after assessment of the left-sided wound healing.

**Fig. 3:**
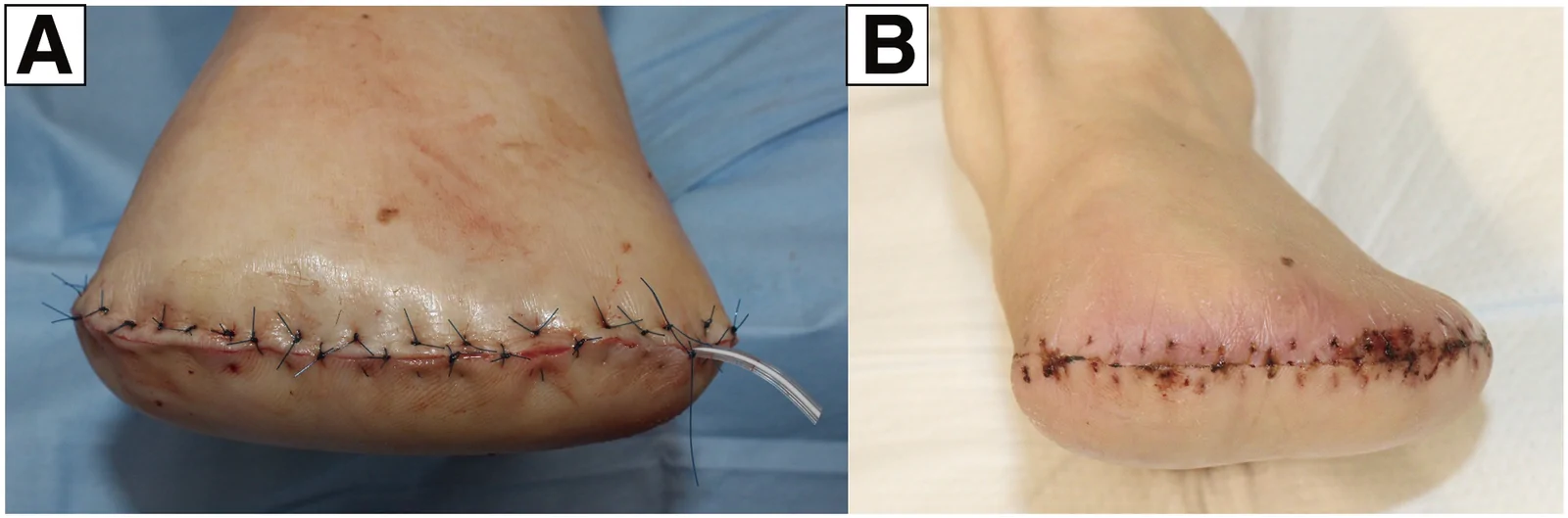
Post-amputation images. (**A**) Image of the left lower limb following transmetatarsal amputation. (**B**) Image of the left lower limb on postoperative day 18.

Angiography performed after 8 sessions of LDL apheresis revealed improved visualization of the dorsal metatarsal arteries and wound blush, improved blood flow velocity, and earlier venous return compared with the baseline angiography (**Fig. 2E**), which showed poor distal perfusion with minimal wound blush and delayed venous return (**Supplementary Video 2A** and **2B**). Video recordings were obtained using the GOKO Bscan-ZD system, enabling the visualization and evaluation of the microcirculation at the dorsalis pedis of the left lower extremity (**Supplementary Video 3A** and **3B**). The GOKO Bscan-ZD examination was performed with the probe placed on the dorsum of the foot (**Supplementary Fig. 1**). Ultrasound coupling gel was applied to ensure adequate contact between the probe and the skin during image acquisition. The microcirculatory velocity in the video recording obtained with the GOKO Bscan-ZD prior to LDL apheresis was 18.1 μm/s (**Supplementary Video 3A**), compared with 169.5 μm/s after 16 sessions of LDL apheresis (**Supplementary Video 3B**). After LDL apheresis, the SPP values at the left dorsalis pedis and plantar region were 24 and 52 mmHg, respectively. The SPP value did not show consistent improvement from that upon admission; however, based on the angiographic findings, favorable vascular changes were observed after LDL apheresis in this case. A transmetatarsal amputation was subsequently performed by a plastic surgery team, and complete wound healing was achieved (**Fig. 3A** and **3B**).

The right lower extremity, which had been managed conservatively during the index hospitalization, was treated during a subsequent admission. The patient underwent a transmetatarsal amputation of the right foot, and complete wound healing was subsequently achieved.

## Discussion

We report herein a case of a refractory ulcer caused by ischemic lesions of the below-the-ankle (BTA) arteries; however, wound healing was ultimately achieved after LDL apheresis and a subsequent transmetatarsal amputation. An increase in microcirculatory flow was observed after LDL apheresis on angiography and in GOKO Bscan-ZD videos. To the best of our knowledge, after a thorough review of the available literature, this is the first report describing the use of GOKO Bscan-ZD to visualize microcirculatory impairment in a patient with CLTI.

In the present case, the feasibility of distal bypass was limited because angiography demonstrated severely impaired pedal circulation and the absence of an adequate distal target vessel. The patency of distal bypass grafts to a severely diseased pedal arch has been reported to be lower than that in patients with intact pedal circulation.^8)^ Therefore, additional surgical revascularization was considered anatomically challenging. EVT is a less invasive vascular reconstruction therapy that can be used for limb salvage in patients with CLTI. Iida et al.^9)^ reported that BTA lesions are predictors of major amputation and delayed wound healing. The reference diameters of the BTA arteries are smaller than those of the proximal BK arteries. A small vessel diameter is a predictor of difficulty in guidewire crossing of BK lesions.^10)^ Moreover, medial calcifications in BTA arteries can indicate revascularization failure.^10)^ In the present case, the target lesion was localized to the BK arteries, particularly in the BTA arteries, with severe calcification. Therefore, achieving adequate revascularization was expected to be difficult. Given the poor wound healing despite EVT and the limited revascularization options, LDL apheresis therapy was selected as an adjunctive treatment aimed at improving microcirculation. This case suggests that LDL apheresis may represent a potential therapeutic option in patients with no-option or limited-option CLTI in whom further surgical or endovascular reconstruction is difficult.

Severity of wounds and target vessels, based on the WIfi classification and the Global Limb Anatomic Staging System scale, are not associated with the efficacy of LDL apheresis.^2)^ LDL apheresis improved SPP values and promoted wound healing in patients with CLTI with BTA lesions.^3)^ However, SPP measurements are susceptible to several factors, including pain during the examination, involuntary movement, cuff positioning, skin condition, and skin temperature, which may limit their reproducibility.^4)^ Furthermore, SPP measurements are more prone to measurement error in patients with severe limb ischemia because markedly impaired skin perfusion can make the measurements technically challenging.^4)^ Because SPP measurements at both baseline and follow-up may have been affected by pain-related motion artifacts and technical limitations, the observed numerical change should be interpreted with caution. Therefore, we cannot determine whether the difference between the 2 measurements reflects a true physiological change or measurement variability. Nevertheless, angiographic findings, GOKO Bscan-ZD assessment, and the subsequent clinical course consistently suggested improved tissue perfusion. Therefore, rather than suggesting superiority over conventional SPP, this case suggests that GOKO Bscan-ZD may serve as a complementary tool for evaluating microcirculation alongside SPP measurements.

Cholesterol crystal embolization (CCE) was also considered in the differential diagnosis because the patient presented with bilateral toe ulcers. However, there was no recent history of catheter-based arterial intervention, no laboratory findings suggestive of CCE, such as renal dysfunction or eosinophilia, and no apparent shaggy aorta on contrast-enhanced CT of the abdominal aorta (**Supplementary Video 1**). Therefore, CCE was considered unlikely, although complement levels were not measured.

The GOKO Bscan-ZD allows for an evaluation of the microvasculature by positioning a specific scope over the sites of interest and measures the velocity of the microcirculation with GOKO-VIP. We believe that this method has the potential to serve as a useful tool for assessing microcirculatory disorders and determining optimal amputation sites. This procedure is not painful or influenced by body movement. Although the probe was placed on the dorsum of the foot in this case, the optimal location for probe placement during these evaluations remains unclear. Moreover, in the present case, serial assessments were not performed during LDL apheresis treatment. Therefore, the temporal course of microcirculatory changes remains unclear, and the findings shown in **Supplementary Video 3** cannot establish that the observed improvement in microcirculation was directly attributable to LDL apheresis. In addition, spontaneous collateral development during the observation period cannot be excluded. Another limitation of this report is that flow velocity measurements were obtained only once before and after treatment. Therefore, measurement reproducibility and intra-observer variability could not be evaluated. Consequently, it remains unclear to what extent the observed increase in flow velocity reflects a true biological change rather than measurement variability. Further studies are warranted to assess the reproducibility and reliability of GOKO Bscan-ZD measurements.

## Conclusion

We report herein a case in which improvement in the microcirculation of a patient with CLTI was observed after LDL apheresis assessed using a GOKO Bscan-ZD device. Although further evidence is required to validate the clinical utility of the GOKO Bscan-ZD, this modality may represent a novel noninvasive method with which to evaluate microcirculation.

## Supplementary Materials

Supplementary Fig. 1 GOKO’s Bscan-ZD capillaroscope. Microcirculation on the dorsum of the foot was observed using a specialized scope. The subject was not the patient described in this case.

Supplementary Video 1 Contrast-enhanced computed tomography from the abdominal aorta to the common femoral artery. No apparent shaggy aorta or severely atheromatous plaques were observed.

Supplementary Video 2 (**A**) Baseline angiography of the dorsalis pedis prior to LDL apheresis. (**B**) Digital subtraction angiography showing improved blood flow velocity, enhanced wound blush, and earlier venous return after 8 sessions of LDL apheresis.

Supplementary Video 3 (**A**) Video recorded using the GOKO Bscan-ZD prior to LDL apheresis. (**B**) Video recorded using the GOKO Bscan-ZD after 16 sessions of LDL apheresis.
