# Supplementary material for: Visualization of Microcirculation Using GOKO Bscan-ZD in a Patient with Chronic Limb-Threatening Ischemia: A Case Report
Source: Ann Vasc Dis. 2026 Aug 28;19(1):26-00061. doi: 10.3400/avd.cr.26-00061 (PMC13533642; doi:10.3400/avd.cr.26-00061)
Supplement: Supplementary file 1 [file avd-19-1-26-00061-s001.pdf]

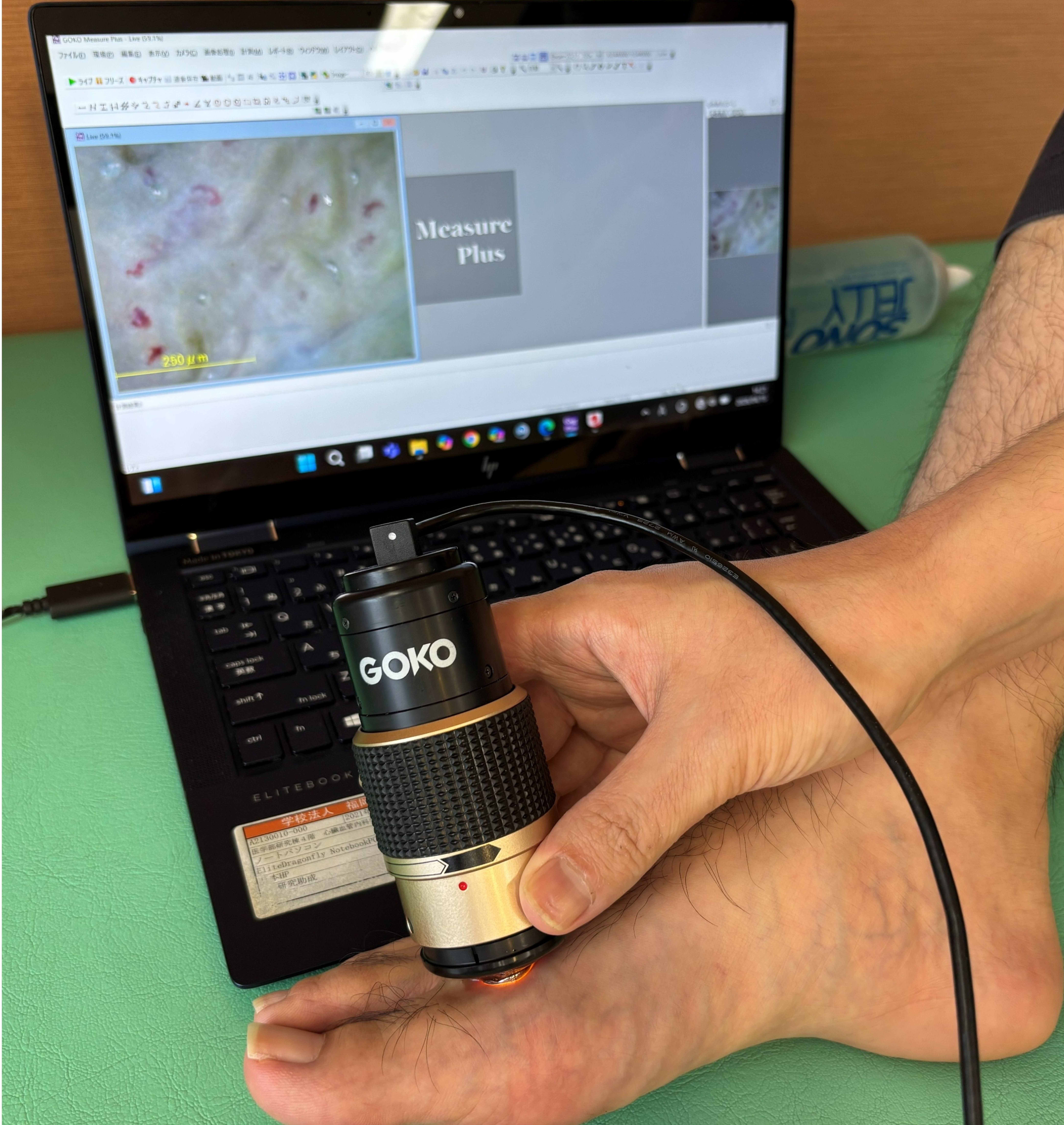
Measure
Plus
250 μm
GOKO
ELITEBOOK
